# Evaluation of the utility value of three diagnostic methods in the detection of malaria parasites in endemic area

**DOI:** 10.1186/s12936-017-1838-4

**Published:** 2017-05-06

**Authors:** Uchenna Iyioku Ugah, Moses Nnaemeka Alo, Jacob Oluwabusuyi Owolabi, Oluchi DivineGift Okata-Nwali, Ifeoma Mercy Ekejindu, Nancy Ibeh, Michael Okpara Elom

**Affiliations:** 1 Department of Microbiology, Faculty of Science, Federal University Ndufu-Alike Ikwo, PMB 1010, Abakaliki, Ebonyi State Nigeria; 2 Department of Medical Laboratory Science, Faculty of Health Sciences and Technology, Nnamdi Azikiwe University, Awka, Nigeria; 3 Department of Medical Laboratory Science, Faculty of Health Sciences and Technology, Ebonyi State University, Abakaliki, Nigeria

**Keywords:** Malaria, RDT, Microscopy, Diagnosis, PCR

## Abstract

**Background:**

Malaria is a debilitating disease with high morbidity and mortality in Africa, commonly caused by different species of the genus *Plasmodium* in humans. Misdiagnosis is a major challenge in endemic areas because of other disease complications and technical expertise of the medical laboratory staff. Microscopic method using Giemsa-stained blood film has been the mainstay of diagnosis of malaria. However, since 1993 when rapid diagnostic test (RDT) kits were introduced, they have proved to be effective in the diagnosis of malaria. This study was aimed at comparing the accuracy of microscopy and RDTs in the diagnosis of malaria using nested PCR as the reference standard. Four hundred and twenty (420) venous blood specimens were collected from patients attending different General Hospitals in Ebonyi State with clinical symptoms of malaria. The samples were tested with Giemsa-stained microscopy and three RDTs. Fifty specimens were randomly selected for molecular analysis.

**Results:**

Using different diagnostic methods, the prevalence of malaria among the subjects studied was 25.95% as detected by microscopy, prevalence found among the RDTs was 22.90, 15.20 and 54.80% for Carestart, SD Bioline PF and SD Bioline PF/PV, respectively. Molecular assay yielded a prevalence of 32%. The major specie identified was *Plasmodium falciparum;* there was co-infection of *P. falciparum* with *Plasmodium malariae* and *Plasmodium ovale.* The sensitivity and specificity of microscopy was 50.00 and 70.59% while that of the RDTs were (25.00 and 85.29%), (25.00 and 94.12%) and (68.75 and 52.94%) for Carestart, SD Bioline PF and SD Bioline PF/PV, respectively. Cohen’s kappa coefficient was used to measure the level of agreement of the methods with nested PCR. Microscopy showed a moderate measure of agreement (k = 0.491), Carestart showed a good measure of agreement (k = 0.611), SD Bioline PF showed a fair measure of agreement (k = 0.226) while SD Bioline PF/PV showed a poor measure of agreement (k = 0.172).

**Conclusions:**

This study recommends that the policy of malaria diagnosis be changed such that the routine diagnosis of malaria is done by a combination of both microscopy and a RDT kit of high sensitivity and specificity so as to complement the errors associated with either of the methods. The finding of *P. ovale* in the study area necessitates an expanded molecular epidemiology of malaria within the study area.

## Background

Malaria is the most common cause of outpatient visits in all age categories in Nigeria and it is the major cause of morbidity and mortality in all parts of Nigeria [1]. It is the second leading cause of death from infectious diseases in Africa, second only to human immunodeficiency virus/acquired immune syndrome (HIV/AIDS) [2]. Malaria is responsible for more hospital cases and deaths than in any other country in the world [2]. It is the largest killer of children under the age of 5 years and has been estimated to kill 3000 children daily [3]. The disease poses a great threat to pregnant women and their fetus; it can result in malarial anaemia and low birth weight. It also hinders cognitive and social development of children and may cause physical and mental impairment in children who survive severe malaria attack [4].

The diagnosis of malaria requires careful clinical examination and laboratory investigation [5]. The microscopic examination of Giemsa-stained thick and thin blood films has been used for the laboratory diagnosis of malaria for more than 100 years [6]. The limitation of microscopy prompted research and development of reliable, easy-to-perform rapid diagnostic tests (RDTs) to detect the presence of malaria parasites at levels of accuracy compared to skilled microscopists [7]. The RDTs detect antigens, such as histidine rich protein 2 (HRP2), *Plasmodium* lactate dehydrogenase (pLDH), or aldolase, which are usually produced during the erythrocytic cycle [5]. At present, different RDTs are available for malaria diagnosis. The performance varies with the environment it is employed in, the geographical location, the disease prevalence and the prevalent parasite species. The sensitivity of RDTs decline with low parasite densities <300–500 μl [7]. The successful implementation of RDT has been bedeviled by poor product performance, inadequate methods to determine the quality of products and a lack of emphasis and capacity to deal with these [8].

Molecular tools are highly sensitive in detecting low levels of infections and accurately detecting species of malaria parasites [9]. Many of these molecular tools for malaria diagnosis range from conventional PCR-based assays, real-time PCR assay, to isothermal assays [10].

The accurate diagnosis of infections by *Plasmodium* species is essential for the reduction of malaria and for epidemiological studies. The World Health Organization (WHO) has recommended microscopy as the gold standard for the diagnosis of malaria. However, the detection of malaria parasites using microscopy requires intensive training of medical laboratory scientists because the result is subjective and requires expertise. It has a very low sensitivity when performed by poorly trained personnel in endemic areas, especially in primary and secondary healthcare facilities [11, 12]. This may result in over diagnosis or under diagnosis with the consequent excessive use of anti-malarial drugs or negligence of treatment which invariably contributes to the increased development of resistance by the malaria parasites, as well as drug-induced pathological conditions. It is, therefore, necessary to assess the current methods used in malaria diagnosis with the aim of making recommendations on the most sensitive and specific method within South-Eastern Nigeria, taking into cognizance the endemic nature, cutting across age barrier and challenges in pregnancy. This will reduce over diagnosis and/or under diagnosis with their associated negative public health implications. Also, the accurate diagnosis of malaria is an essential aspect in the reduction of malaria morbidity, drug resistance intensity and malaria control.

This comparative study on malaria diagnostic methods is aimed at providing an evidence-based criteria on diagnostic options in malaria endemic areas, when critical decision concerning treatment is required that will provide a high level of specificity and sensitivity.

## Methods

### Study area and design

This study was carried out in Ebonyi State, Southeastern Nigeria. Samples were collected from the general hospitals in Ebonyi State. It is a double-blind clinical diagnostic assay of malaria using microscopy and three RDT kits and comparing their results against that of nested PCR, as the gold standard.

### Study population

Simple random sampling technique was used to select the subjects recruited for the study. The study populations comprised of individuals who had shown clinical signs of malaria and in whom test for malaria parasite have been requested for by clinicians and who assented to participate in the study. The subject population included persons of different age groups such as children, adults and pregnant women.

### Specimen collection

Peripheral blood samples were collected by finger prick and by venepuncture. The finger prick samples were collected with sterile lancet after cleaning the finger with 70% alcohol. Three drops of blood from the finger were used to impregnate dry blood spot (DBS) papers (Lasec, S.A). A total of six DBS spots were made on each DBS paper for each specimen. These specimens were preserved for molecular analysis. 2 ml of venous blood specimens were also collected by venepuncture using sterile syringe and needles. The specimens were dispensed into EDTA containers and immediately used to make the thin and thick films as well as the RDT tests.

### Laboratory analysis

The subjects were screened for malaria parasites using Giemsa-stained malaria microscopy, three RDT kits, among the specimens collected, a total of fifty (50) were randomly selected for nested polymerase chain reaction.

### Microscopy

Microscopy was performed to detect malaria parasites and the species were identified based on morphological features. Thick and thin film of the peripheral blood specimens were made immediately after collection on a clean, grease-free microscope slide and allowed to air dry on the laboratory bench. The films were stained with 10% Giemsa solution (Sigma-Aldrich, USA) for 30 min, allowed to air dry and subsequently examined using oil immersion objective lens and all the fields were examined before declaring a slide negative. For each specimen, the thick films were examined first for detection of malaria parasites, the thin films of each specimen were subsequently examined for speciation only in those specimens in which malaria parasites were identified in the thick film.

The slides were examined by two malaria microscopists who are certified by the WHO. Each microscopist examined each specimen independently and results were recorded as being positive when both microscopists recorded a positive result.

### Rapid diagnostic test

Three different commercially available RDT kits for malaria parasites having different sensitivity and specificity were used to detect malaria parasites in the blood samples. These are; Carestart™ (Access Bio Inc. New Jersey, USA) with Batch Numbers MO2K2, SD Bioline PF (Biostandard Diagnostics Inc. Gurgaon, Korea) with Batch Numbers 082352 and SD Bioline PF/PV (Biostandard Diagnostics Inc. Gurgaon, Korea) with Batch Numbers 05AD14038. The tests were performed by strictly adhering to the manufacturers’ instructions.

The test device was removed from the foil pouch and placed on a flat, dry surface. 5 µl of whole blood was collected with the capillary pipette provided and transferred to the round sample well. Four drops of the assay diluents was added vertically into the square assay diluent well. Results were read after 15 min over a bright light.

### Molecular assay

A dry blood spot was made from each of the 50 selected blood samples on a DBS paper (Lasec, S.A) for PCR assay. Three drops of the blood from finger prick were used to impregnate 3 mm DBS papers (Lasec, S.A) and allowed to air dry on a paper rack. Each filter paper specimen was stored in a plastic bag at room temperature with a desiccator and transported to the laboratory for molecular assay [13]. Molecular assay was done at Nigerian Institute of Medical Research (NIMR) Yaba, Lagos, Nigeria.

Molecular analysis using nested PCR technique based on amplification of 18s rRNA genes as described by Fuehrer et al. [14] was used to detect the genes for *Plasmodium* genus, in the second reaction the genes for *Plasmodium* species were identified using methods described by Snounou et al. [15].

### DNA extraction

DNA extraction was done using the Saponin-Chelex extraction method. Saponin (0.5%) in phosphate buffered saline (PBS) was prepared by dissolving 1 g of saponin and one tablet of PBS (Sigma-Aldrich, USA) in 200 ml of sterile distilled water, shaken vigorously and allowed to stand till the saponin and PBS dissolved completely. Each of the dried blood spot was cut out from the DBS papers with sterilized scissors with serrated edges and each of the discs were transferred to pre-labelled Eppendorf tubes. 1 ml of the saponin-PBS solution was added to the specimen in the Eppendorf tubes and allowed to stand for 24 h at 4 °C for complete haemolysis.

The specimens were washed three times in PBS with intermittent centrifugation at 13,000 rpm for 5 min after each wash. Subsequently, 1 ml of PBS was added to the sediment, and then it was vortexed for 1 min and incubated for 30 min at 4 °C. It was centrifuged again for 5 min at 13,000 rpm and the supernatant decanted.

Fifty microlitres of freshly prepared Chelex solution was added to the tubes followed by 150 μl of distilled water to obtain 5% of the Chelex solution. It was then vortexed and incubated in a block heater set at 99 °C for 20 min, centrifuged at 13,000 rpm for 5 min and the supernatants decanted into newly labelled Eppendorf tubes. The supernatants contain the DNA.

### Assessment of DNA yield and purity

DNA yield was measured using a nanodrop spectrophotometer (NANO 1000, China) based on maximum absorbance of DNA at 260 nm. 1μl of the DNA sample was applied on the platform of the nanodrop spectrophotometer and a reading was taken after adjustment of absorbance to zero using water as blank. The yield was measured in ng/μl. The 260/280 nm ratio was obtained to give an analysis of the purity of the sample and the concentration of the extracted DNA was also found.

### Polymerase chain reaction

PCRs were performed according to Snounou et al. [15] nested-protocol with some minor modifications. All PCR amplifications (TC 312 Thermocycler, Techne, USA) were performed in 20 μl volume containing 1.5 mM MgCl2, 0.12 mM dNTPs, 240 nM of each oligonucleotide and 0.5 U of *Taq* polymerase (Invitrogen^®^, USA). A species-specific region of the 18 rDNA of *Plasmodium* was amplified (All the primers used were synthesized by Inqaba biotec S.A). In the first reaction, 10 μl volume equivalent of extracted DNA was added, using pairs of primers targeting an outer region specific to the *Plasmodium* genus. One microliter of the first reaction product was then used as a template in a second nested reaction to yield specific *Plasmodium falciparum, Plasmodium malariae* and *Plasmodium ovale* products.

The temperature profile for the PCR was: 5 min at 95 °C; 25 cycles of 1 min at 94 °C, 2 min at 58 °C, 2 min at 72 °C; followed by the second reaction where the annealing temperature was modified to 65 °C and the cycles repeated 30 times.

The PCR products were visualized under UV light on 2% agarose gel after electrophoresis in 0.5X Tris borate EDTA buffer and ethidium bromide staining. A sample was considered positive if a 205 or 125 or 105 bp products for *P. falciparum, P. malariae* and *P. ovale* was detected. In every set of reactions, negative (PBS) and positive controls for *P. falciparum, P. malariae* and *P. vivax* (3D7 Pf) were used. The primary reaction yielded a 500 bp PCR product.

### Data analysis

Data was analysed using descriptive statistics, Cohen’s kappa coefficient and student’s *T* test. Cohen’s kappa coefficient was used to compare the measure of agreements between microscopy and the RDTs versus nested PCR results as the reference standard. All statistical analysis were calculated at 95% level of significance. The sensitivity, specificity, positive predictive value and negative predictive value were calculated using the formulae presented below. Data analysis was performed with the aid of statistical programme for social sciences (SPSS) version 18.0

$${\text{Sensitivity}}\,{ = }\,\frac{\text{TP}}{{ ( {\text{TP}}\,{ + }\,{\text{FN)}}}} \, \times { 100}\,$$

$${\text{Specificity}}\,{ = }\,\frac{\text{TN}}{{ ( {\text{TN}}\,{ + }\,{\text{ FP)}}}} \, \times { 100}$$

$${\text{Positive predictive value}}\,{ = }\,\frac{\text{TP}}{{ ( {\text{TP}}\,{ + }\,{\text{FP)}}}} \, \times { 100}$$

$${\text{Negative predictive value}}\,{ = }\,\frac{\text{TN}}{{ ( {\text{TN}}\,+\,{\text{FN)}}}} \, \times { 100}$$

Key: TP is true positive, FN is false negative, TN is true negative, FN is false negative.

## Results

A total of four hundred and twenty (420) subjects were enlisted for this study. The subjects comprised of 255 females (60.70%) and 165 males (39.30%), having an age range of 2–65 years with a mean age of 34 years. Malaria parasite was detected in 109 (25.95%) of the subjects by microscopy and all the species detected by microscopy were observed to be *P. falciparum.* Among the subjects studied, 96 (22.9%), 64 (15.2%) and 230 (54.8%) were positive by Carestart, SD Bioline PF and SD Bioline PF/PV respectively (Table 1).

**Table 1:** Comparison of the sensitivity, specificity, positive predictive value, negative predictive value and kappa values of microscopy and RDTs versus PCR

Method	Sensitivity (95% CI)	Specificity (95% CI)	PPV (95% CI)	NPV (95% CI)	Kappa
Microscopy	50.00 (26.45–75.35)	70.59 (52.52–84.90)	44.44 (21.53–69.24)	75.00 (56.60–88.54)	0.491
Carestart	25.00 (7.27–52.38)	85.29 (68.94–95.05)	44.44 (13.70–78.80)	70.73 (54.46–83.87)	0.611
SD Bioline PF	25.00 (7.27–52.38)	94.12 (80.32–99.28)	66.67 (22.28–95.67)	72.73 (57.21–85.04)	0.226
SD Bioline PF/PV	68.75 (41.34–88.98)	52.94 (35.13–70.22)	40.74 (22.39–61.20)	78.26 (56.30–92.54)	0.172

*PPV* positive predictive value, *NPV* negative predictive value, *CI* confidence interval

Based on the results obtained from microscopy, males had a prevalence of 25.45% (42 of 165) while females had a prevalence of 26.27% (Table 2). Among the positive cases, the highest prevalence was observed among the age range of 31–35 with a prevalence of 34.43% (21 of 61). This age group had the highest overall prevalence among the positive cases with a prevalence of 19.27% (21 of 109). A total of 18 under five children were studied, and a prevalence of 33.33% was found. Among the pregnant women studied, a total of 62 subjects were pregnant, among which 19 (30.65%) were positive while 43 (69.35%) were negative.

**Table 2:** Prevalence of malaria among the age groups by microscopy

Age range	Positive (%)	Negative (%)	Total (100%)
0–5	06 (33.33)	12 (66.67)	18
6–10	05 (26.32)	14 (73.68)	19
11–15	02 (15.38)	11 (84.62)	13
16–20	02 (25.00)	06 (75.00)	08
21–25	05 (19.23)	21 (80.77)	26
26–30	17 (22.37)	59 (77.63)	76
31–35	21 (34.43)	40 (65.57)	61
36–40	13 (18.31)	58 (81.69)	71
41–45	19 (29.23)	46 (70.77)	65
46–50	08 (25.81)	23 (74.19)	74
51–55	07 (33.33)	14 (66.67)	21
56–60	02 (40.00)	03 (60.00)	05
61–65	02 (33.33)	04 (66.67)	06
Total	109 (25.95)	311 (74.05)	420

Among the specimens that were subjected to molecular analysis, 16 (32.0%) were positive while 34 (68.0%) were negative for *Plasmodium* genes. *Plasmodium falciparum* was observed in all positive cases, *P. malariae* was present in five (5 of 16) cases while *P. ovale* was present in one (1 of 16) subject. Both *P. malariae* and *P. ovale* were present as co-infection with *P. falciparum* in all the subjects they were found.

Cohen’s kappa coefficient was used to compare the agreement between microscopy and rapid diagnostic tests using PCR results as the gold standard. This study found that when compared between microscopy and PCR, there was moderate agreement (k = 0.491). Among the RDT kits, Carestart showed good agreement with PCR (k = 0.611), SD Bioline PF showed fair agreement (k = 0.226) while SD Bioline PF/PV showed poor agreement (k = 0.172).

When the agreements between the RDTs and microscopy were compared, Carestart and microscopy had a fair agreement (k = 0.233), whereas SD Bioline PF and SD Bioline PF/PV had poor agreements with microscopy (k = 0.192) and (k = 0.085), respectively.

When the agreements between the RDTs were compared, SD Bioline PF and SD Bioline PF/PV had poor agreement (k = 0.169), Carestart and SD Bioline PF/PV had fair agreement (k = 0.257) while Carestart and SD Bioline PF had a moderate agreement (k = 0.465).

## Discussion

Molecular methods have generally been accepted to offer excellent sensitivity and specificity and are considered as reference standard for diagnosis of malaria infection [16]. This study used nested polymerase chain reaction result as the gold standard to compare the results obtained from Giemsa-stained microscopy and three rapid diagnostic test kits after the tests were performed independently of each other.

This study observed generally reduced malaria prevalence in the different methods employed. This finding is consistent with that of other studies [17–19]. It is an indication that there is a reduction in the prevalence and burden of malaria in the study area.

The low prevalence of malaria among under five children and pregnant women observed in this study is an indication of the positive results of malaria control and prevention programmes, such as free treatment of malaria for these population groups and administration of anti-malarial drugs to pregnant women in antenatal clinics, the introduction of ACT anti-malarial drugs in the treatment of malaria, as well as the sustenance of intermittent preventive therapy (IPT) during pregnancy. It therefore calls for more intensive effort to be geared towards malaria eradication.

Different intervention strategies are currently in place to reduce the burden of malaria. However, in spite the low political commitment to these intervention strategies, the overall widespread mass education/awareness about preventive practices through the mass media have contributed immensely in reducing malaria prevalence among the population.

Microscopy yielded a prevalence of 25.95% (109 of 420), with a moderate measure of agreement (k = 0.491) when compared with PCR result. This finding is not in consonance with the good measure of agreement with nested PCR observed by Alemu et al. [20]. Microscopy is subjective and the result obtained is dependent on the quality of training of the observer. In this study, two malaria microscopists who are certified by the WHO studied each specimen independently and results were recorded as being positive when both microscopists recorded a positive result. The difference found by this study and that of Alemu et al. [20] may be due to the subjective nature of results obtained by malaria microscopy and this further makes it necessary to review the current WHO policy, which requires that malaria diagnosis be established by malaria microscopy. It also calls for more training of laboratory staff on malaria microscopy. The moderate measure of agreement found in this study between microscopy and PCR underscores the need to include another method as a backup check in the routine diagnosis of malaria in all healthcare laboratory facilities.

The sensitivity and specificity of microscopy when compared with the nested PCR method is in contrast to the study by Alemu et al. [20], which found higher values of sensitivity and specificity (74.0 and 87.4%), respectively. The sensitivity of microscopy is limited to approximately 20 parasites/µl of blood and subjective interpretation as well as reader error contributes to the reduction in the accuracy of diagnosis [20]. However, in spite of the inherent limitations of malaria Giemsa microscopy, the quality of diagnosis largely depends on the quality of training of the microscopist. Adequate training can increase the yield of accurate malaria diagnosis which helps to reduce illness, potential death, mistreatment and persistently high disease burden while at the same time saving vital resources for malaria control [21]. The responsibility however resides with the management of healthcare facilities to ensure the routine periodic training and retraining of their staff on malaria microscopy to ensure that microscopy results are of high sensitivities, specificities and accuracy.

In this study, malaria microscopy only detected *P. falciparum* in some specimens which had co-infection of *P. falciparum* with either *P. malariae* or *P. ovale.* This occurrence was also observed in other studies by Osman et al. [22] and Khan et al. [23] that also reported failure in detecting missed co-infections by microscopy when compared with results obtained by PCR. This observation may be attributed to human errors of the microscopist and this is one of the limitations of microscopy and necessitates the addition of another diagnostic method to ensure the standardization of malaria test results across different laboratories and hence accentuate quality assurance. This further re-inforces the need for periodic training and retraining of the laboratory scientist on malaria microscopy to ensure increased accuracy of malaria microscopy result.

Nested PCR utilizes genus and species specific markers for the detection of *plasmodium* parasites. This allows for the detection of low density infections as well as mixed infections which are routinely missed in microscopy and makes nested PCR an ideal confirmatory test for malaria diagnosis [20]. The results obtained from this study are consistent with those of other studies from Africa, Asia and Latin America reporting a considerably higher potential for detection of mixed infections [20, 24–26].

This study found the presence of *Plasmodium ovale* and this finding has not been reported by other studies which had been conducted among the study population. This might be attributed to the absence of molecular techniques in previous studies which used either microscopy or RDTs.

Although microscopy is still considered the gold standard for the diagnosis of malaria, many countries consider shifting their diagnostic policy for malaria to implementing RDTs as standard diagnosis. This is because in many areas, proper microscopy is not possible because of lack of electricity or equipment [22]. Malaria RDTs of good quality can, when appropriately used provide a rapid and reliable way to demonstrate the presence or absence of malaria parasites at all levels of health service. Hence, RDTs are considered a good alternative for microscopy but owing to its own limitations such as poor storage conditions, low quality, availability and economic cost, needs to be carefully considered when implemented.

The three RDTs studied showed different measures of agreements with PCR. Good measure (k = 0.611), fair measure (k = 0.226) and poor measure (k = 0.172) was observerd for Carestart, SD Bioline PF and SD Bioline PF/PV respectively. Besides HRP2 gene deletion which may be responsible for the varying accuracies observed among RDTs, They are affected by extraneous factors such as storage and handling. Most RDTs are not produced in Nigeria hence the climate may affect the efficacy of these RDTs. Also, in countries like Nigeria, RDTs are sold by traders, most of who know little or nothing about the influence of environmental storage conditions on the accuracy of the results. Care must be taken to ensure that when RDTs are purchased in large quantities, storage conditions prescribed by the manufacturers are strictly followed to ensure that the potency of the test kits are adequately preserved and hence assure the quality of results obtained by their use.

RDTs in conjunction with microscopy should improve the diagnosis of malaria. However, RDT use should be considered as more cost—effective in the areas characterized by high or moderate intensity malaria transmission and in situations where health services are deficient or absent such as primary health centers [27]. In developing countries which are malaria endemic areas, there are several other factors that affect the result of malaria microscopy such as skill of the personnel which may compromise the specificity of malaria microscopy hence the need to include RDT in routine malaria investigations in endemic areas as this will improve the accuracy of detection to malaria parasites from patients in real time situations.

This study was carried out in an endemic area where technical expertise are deficient as found in many developing countries therefore a combination of microscopy and RDT will surely enhance the sensitivity and specificity of malaria diagnosis in both clinical and research settings.

The finding that RDTs had low sensitivities (25.00, 25.00 and 68.75%) for Carestart, SD Bioline PF and SD Bioline PF/PV respectively is also comparable to the study by Kashif et al. [28] who reported lower sensitivities of the RDTs tested than microscopy. However the specificities of the RDTs tested were higher (85.29, 94.12 and 52.94%), respectively.

**Fig. 1:**
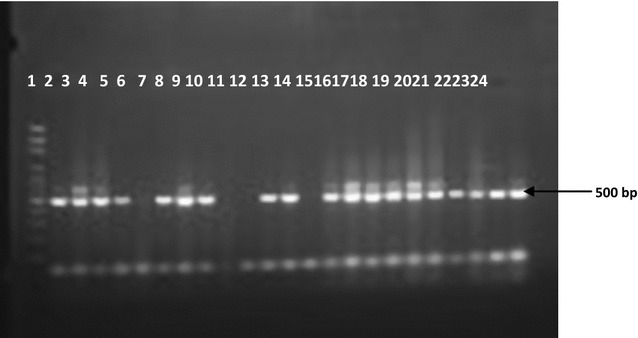
Agarose gel electrophoresis of the human plasmodium 18s rRNA gene PCR product. *Lane 1* = 100 bp DNA marker; *Lane 2* = *P. falciparum* 3D7 control; *Lanes 3–24* = *Plasmodium* infected blood DNA samples

**Fig. 2:**
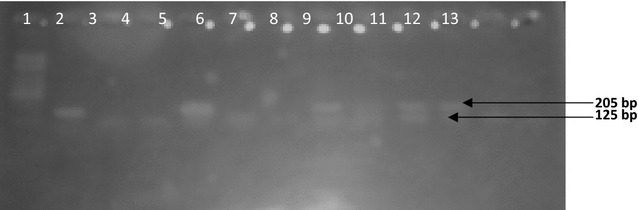
Agarose gel electrophoresis of the nested PCR products for human *Plasmodium* species using species-specific primers. *Lane 1* = 100 bp DNA marker; *Lane 2* = *P. falciparum* 3D7 control; 205 bp product = *P. falciparum*; 125 bp product = *P. malariae*

The low sensitivity and high specificity observed in all the test kits studied is in concordance with that of other studies [29–31]. The high negative predictive values obtained in this study, (75.00, 70.73, 72.73 and 78.26%) for microscopy, Carestart, SD Bioline PF and SD Bioline PF/PV respectively allows for confirmatory diagnosis of negative test patients as non-malaria patients, hence there is reduced risk of missing an infected patient (Figs. 1, 2).

## Conclusion

This study has found microscopy still being a good method for the diagnosis of malaria and having a good measure of agreement with PCR. Hence it is recommended that the laboratory diagnosis of malaria be performed using both microscopy and RDT of high sensitivity and specificity. The combination of these methods will ensure that laboratory reports on malaria diagnosis are of very high accuracy. Furthermore, the impact of routine training and retraining of medical laboratory staff on malaria microscopy cannot be overemphasized.

## Abbreviations

PCR: polymerase chain reaction
DNA: deoxyribonucleic acid
rDNA: ribosomal deoxyribonucleic acid
UV Light: ultraviolet light
EDTA: ethylenediaminetetraacetic acid

## References

[1] Federal Ministry of Health (2005). National malaria and vector control division.

[2] WHO (2013). Malaria factsheet.

[3] WHO (2012). World malaria report 2012.

[4] Benson J, Philip C, Kay M (2013). Low vitamin B12 levels among newly—arrived refugees from Bhutan, Iran and Afghanistan. A multicentre Australian study. PLoS ONE.

[5] Orogade A, Okwa O (2012). Current issues in clinical and laboratory diagnosis of malaria. Malaria parasites, vol 29.

[6] Moody A (2002). Rapid diagnostic tests for malaria parasites. Clin Microbiol Rev.

[7] Kim SH, Nam MH, Roh KH, Park HC, Nam DH (2008). Evaluation of a rapid diagnostic test specific for *Plasmodium vivax*. Trop Med Int Health.

[8] Bell D, Wongsrichala C, Barnwell J (2006). Ensuring quality and access for malaria diagnosis: how can it be achieved?. Nat Rev Microbiol.

[9] Okell LC, Ghani AC, Lyons E, Drakeley CJ (2009). Submicroscopic infection in *Plasmodium falciparum*-endemic populations: a systemic review and meta-analysis. J Infect Dis.

[10] Lucchi NW, Narayanan J, Karell MA, Xayavong M, Kariuki S, DaSilva AJ (2013). Molecular diagnosis of malaria by photo-induced electron transfer fluorogenic primers: PET-PCR. PLoS ONE.

[11] Coleman RE, Maneechai N, Rachaphaew N, Kumpitak C, Miller RS (2002). Comparison of field and expert laboratory microscopy for active surveillance for asymptomatic *Plasmodium falciparum* and *Plasmodium vivax* in Western Thailand. Am J Trop Med Hyg.

[12] Bates I, Bekoe V, Asamoa-Adu A (2004). Improving the accuracy of malaria-related tests in Ghana. Malar J.

[13] Dahlstrom S, Veiga MI, Ferreira P, Martensson A, Kaneko A, Andersson B (2008). Diversity of the sarco/endoplasmic reticulum Ca(2+)-ATPase orthologue of *Plasmodium falciparum* (PfATP6). Infect Genet Evol.

[14] Fuehrer HP, Fally MA, Habler VE, Starzengruber P, Swoboda P, Noedl H (2011). Novel nested direct PCR technique for malaria diagnosis using filter paper samples. J Clin Microbiol.

[15] Snounou G, Viriyakasol S, Jarra W, Thaithong S, Brown KN (1993). Identification of the four human malaria parasite species in field samples by the polymerase chain reaction and detection of a high prevalence of mixed infections. Mol Biochem Parasitol.

[16] Bourgeois N, Boulet A, Bousquet PJ, Basset D, Douard-Enault C, Charachon S (2010). Comparison of three real-time PCR methods with blood smears and rapid diagnostic test in *Plasmodium* species infection. Clin Microbiol Infect.

[17] Kim D, Fedak K, Kramer R (2012). Reduction of malaria prevalence by indoor residual spraying: a meta-regression analysis. Am J Trop Med Hyg.

[18] Bradley J, Rehman AM, Schwabe C, Vargas D, Monti F, Ela C (2013). Reduced prevalence of malaria infection in children living in houses with window screening or closed eaves in Bioko Island, Equatorial Guinea. PLoS ONE.

[19] Bedu-Addo G, Gai PP, Meese S, Eggelte TA, Thangaraj K, Mockenhaupt FP (2014). Reduced prevalence of placental malaria in primiparae with blood group O. Malar J.

[20] Alemu A, Fuehrer HP, Getnet G, Kassu A, Getie S, Noedl H (2014). Comparison of Giemsa microscopy with nested PCR for the diagnosis of malaria in North Gondar, North-west Ethiopia. Malar J.

[21] Zurovac D, Larson BA, Akhwale W, Snow RW (2006). The financial and clinical implications of adult malaria diagnosis using microscopy in Kenya. Trop Med Int Health.

[22] Osman MM, Nour BY, Sedig MF, De Bes L, Babikir AM, Mohamedani AA (2010). Informed decision-making before changing to RDT: a comparison of microscopy, rapid diagnostic test and molecular techniques for the diagnosis and identification of malaria parasites in Kassala, Eastern Sudan. Trop Med Int Health.

[23] Khan SA, Ahmed S, Mushahid N, Anwer M, Saeed S, Khan FA (2013). Comparison of real time polymerase chain reaction with microscopy and antigen detection assay for the diagnosis of malaria. J Coll Physicians Surg Pak.

[24] Rudolfo H, De Donato M, Mora R, Gonzalez L, Contreras CE (2007). Comparison of the diagnosis of malaria by microscopy, immunochromatography and PCR in endemic areas of Venezuela. Braz J Med Biol Res.

[25] Wangai LN, Karau MG, Njiruh PN, Sabah O, Kimani FT, Mayoma G (2011). Sensitivity of microscopy compared to molecular diagnosis of *P. falciparum*: implications on malaria treatment in epidemic areas in Kenya. Afr J Infect Dis.

[26] Abdel-Wahab MM, Ismail KA, El-Sayed NM (2012). Laboratory diagnosis of malaria infection in clinically suspected cases using microscopic examination, optimal rapid antigen test and PCR. Parasitol United J.

[27] Nicastri E, Bevilacqua N, Schepisi MS, Paglia MG, Meschi S, Ame SM (2009). Accuracy of malaria diagnosis by microscopy, rapid diagnostic test and PCR methods and evidence of antimalarial overprescription in non-severe febrile patients in two Tanzanian hospitals. Am J Trop Med Hyg.

[28] Kashif AH, Adam GK, Mohammed AA, Elzaki SE, AbdelHalim AM, Adam I (2013). Reliability of rapid diagnostic test for diagnosing peripheral and placental malaria in an area of unstable malaria transmission in Eastern Sudan. Diagn Pathol.

[29] Paris DH, Imwong M, Faiz AM, Hasan M, Yunus EB, Silamut K (2007). Loop-mediated isothermal PCR (LAMP) for the diagnosis of falciparum malaria. Am J Trop Med Hyg.

[30] Ishengoma DS, Francis F, Mmbando BP, Lusingu JPA, Magistado P, Alifrangis M (2011). Accuracy of malaria rapid diagnostic tests in community studies and their impact on treatment of malaria in an area with declining malaria burden in north-eastern Tanzania. Malar J.

[31] Singh R, Abdullahi K, Bunza MDA (2013). Comparative diagnosis of falciparum malaria infections by microscopy, two RDTs and Nested PCR in the three states of North-western Nigeria. J Biol Agric Healthc.

